# Method Development for Determination of Doripenem in Human Plasma via Capillary Electrophoresis Coupled with Field-Enhanced Sample Stacking and Sweeping

**DOI:** 10.3390/ijms241813751

**Published:** 2023-09-06

**Authors:** Hsin-Hua Liang, Yu-Chao Lin, Chin-Chuan Hung, Yu-Chi Hou, Yi-Hui Lin

**Affiliations:** 1 School of Pharmacy, China Medical University, Taichung 406040, Taiwan; 2 Division of Pulmonary and Critical Care Medicine, Department of Internal Medicine, China Medical University Hospital, Taichung 404332, Taiwan

**Keywords:** carbapenems, doripenem, FESS-sweeping MEKC, human plasma, online preconcentration

## Abstract

In this study, we established a novel capillary electrophoresis method for monitoring the concentration of doripenem in human plasma. As a time-dependent antibiotic, doripenem maximizes its antibacterial effects and minimizes the potential for antibiotic resistance through careful therapeutic drug monitoring. Two online preconcentration techniques, field-enhanced sample stacking (FESS) and sweeping, were coupled to enhance the detection sensitivity. Briefly, an uncoated fused silica capillary (40 cm × 50 μm i.d) was rinsed with a high conductivity buffer (HCB) composed of 150 mM phosphate buffer (NaH_2_PO_4_, pH 2.5) and 20% methanol. A large sample plug prepared in a low-conductivity phosphate buffer (50 mM NaH_2_PO_4_, pH 2.5) was then hydrodynamically injected (5 psi, 80 s) into the capillary. Under an applied voltage of −30 kV, the analyte was accumulated at the FESS boundary and swept by the negatively charged micelles toward the UV detector. Plasma samples were pretreated by solid-phase extraction (SPE) to eliminate endogenous interferences. The validation results demonstrated a high coefficient of determination (r^2^ > 0.9995) for the regression curve with impressive precision and accuracy: relative standard deviation (RSD) <5.86% and relative error <4.63%. The limit of detection (LOD, S/N = 3) for doripenem was determined to be 0.4 μg/mL. Compared to the conventional micellar electrokinetic chromatography method, our developed method achieved a sensitivity enhancement of up to 488-fold for doripenem. Furthermore, the newly developed method successfully quantified doripenem concentrations in plasma samples obtained from patients accepting doripenem regimens, proving its application potential in the clinical realm.

## 1. Introduction

Antibiotic-resistant infections have emerged as a critical public health concern, demanding immediate attention. The World Health Organization (WHO) and the Centers for Disease Control and Prevention (CDC) emphasize the need for improvements in the appropriate use of antibiotics, which is considered a priority issue [1,2]. 

Carbapenem antibiotics represent a final resort for treating multidrug-resistant infections, and their usage has progressively increased as empirical therapy. Unfortunately, this trend has exacerbated the emergence and spread of resistance [3,4]. Among these antibiotics, doripenem stands out for its exceptional efficacy against multidrug-resistant infections, proving effective against both Gram-positive and Gram-negative bacteria, including challenging pathogens like *Pseudomonas aeruginosa* (*P. aeruginosa*) and aerobes [5]. Doripenem belongs to the category of time-dependent antibiotics, for which its in vivo activity is linked to the percentage of time during the dosing interval that the drug concentration remains above the minimum inhibitory concentration (MIC) (%T>MIC) [6]. To combat certain challenging bacterial pathogens (MIC ≤ 2 µg/mL), it is recommended to maintain a plasma drug concentration of at least 40% T>MIC [6,7]. However, clinical outcomes often fall short of expectations due to the variable nature of disease states and patient-specific pharmacokinetic/pharmacodynamic (PK/PD) variability. Doripenem is a highly hydrophilic antibiotic with a low protein binding rate and is mainly excreted by the kidney, with 71% unchanged and 15% as a ring-opened inactive metabolite. For patients with renal impairment or undergoing hemodialysis, dose adjustments are required to reduce or supplement the plasma concentration of doripenem, but clear recommendations to date have been insufficient [5,6]. The suboptimal exposure of antibiotics can lead to treatment failures and exacerbate the threat of carbapenem-resistant infections [8]. Consequently, several clinical interventions can enhance antibiotic efficacy, including extended infusion times, higher dosages, and therapeutic drug monitoring. Prolonged infusion time from 1 h to 4 h was found to maximize the %T>MIC of doripenem [5,9]. The continuous administration of meropenem, employing a higher loading dose and an extended dosing interval than the standard intermittent administration, has demonstrated advantages in clinical practice [10,11]. To sum up, the doripenem concentration in plasma serves as the basis of informed decision making and dosage adjustment, which is closely related to the achievement of a therapeutic effect, the success of antimicrobial stewardship, and the response to the threat of antibiotic resistance [6,7,12,13,14]. Therefore, the development of an efficient analytical method is imperative for accurately determining plasma drug concentrations.

Currently, the quantitative analysis of doripenem in biological fluids primarily relies on high-performance liquid chromatography (HPLC) in combination with either an ultraviolet detector (UV) or mass spectrometry (MS) [15,16,17,18,19]. While these validated methods offer specificity and sensitivity, they require the use of large amounts of solvents, leading to high analysis costs and posing environmental concerns. Capillary electrophoresis (CE) presents itself as a viable alternative. CE exhibits a highly efficient analytical performance and offers advantages such as simplicity of operation, low reagent consumption, high resolution, and adjustable parameters. Though CE suffers from reduced sensitivity due to its short optical path and small sample size, this limitation has been overcome by integrating online preconcentration techniques [20,21]. Four main types of online preconcentration techniques have been widely used and reviewed, including field-enhanced sample stacking (FESS) [22,23], dynamic pH junction [24], sweeping [25], and transient isotachophoresis (tITP) [26]. These techniques successfully enhance sensitivity by concentrating the analytes into narrow zones before reaching the detection window without requiring additional equipment. However, only a few CE methods, mostly applied in formulation analysis, have been developed for the analysis of carbapenems [27,28,29,30,31,32]. Michalska et al. have utilized CE in conjunction with the online preconcentration technique of sweeping to analyze ertapenem and its impurities in pharmaceutical formulations [33]. Their study employed two different modes, namely normal stacking mode (NSM) and stacking with reverse migrating mode (SRMM), for the analysis. They successfully extended the sample injection time from 10 s to 50 s while maintaining a good peak shape. This method resulted in a tenfold increase in analytical sensitivity. No previous examples were found in the literature regarding the application of CE coupled with online preconcentration techniques for the analysis of doripenem.

Our objective was to develop a CE method that is simple, rapid, and highly sensitive for analyzing the concentration of doripenem in human plasma. Before analysis, we implemented solid-phase extraction (SPE) as a sample pretreatment step to eliminate biological interferences. Considering the hydrophilic nature of doripenem, we effectively addressed the extraction challenge by acidifying the samples. However, since there were still significant amounts of hydrophilic substances present, we devised a combined approach of sweeping with field-enhanced sample stacking (FESS) for online preconcentration. To the best of our knowledge, this is the first time a CE method incorporating FESS-sweeping micellar electrokinetic chromatography (FESS-sweeping MEKC) has been utilized to determine the doripenem concentration in human plasma. We successfully achieved the desired sensitivity and selectivity through optimized conditions with this newly established method. It is worth noting that this FESS-sweeping MEKC method has been successfully employed to quantify doripenem concentrations in actual plasma samples, indicating its potential for future applications in clinicals. 

## 2. Results and Discussion

### 2.1. Extraction Process

**Figure 1:**
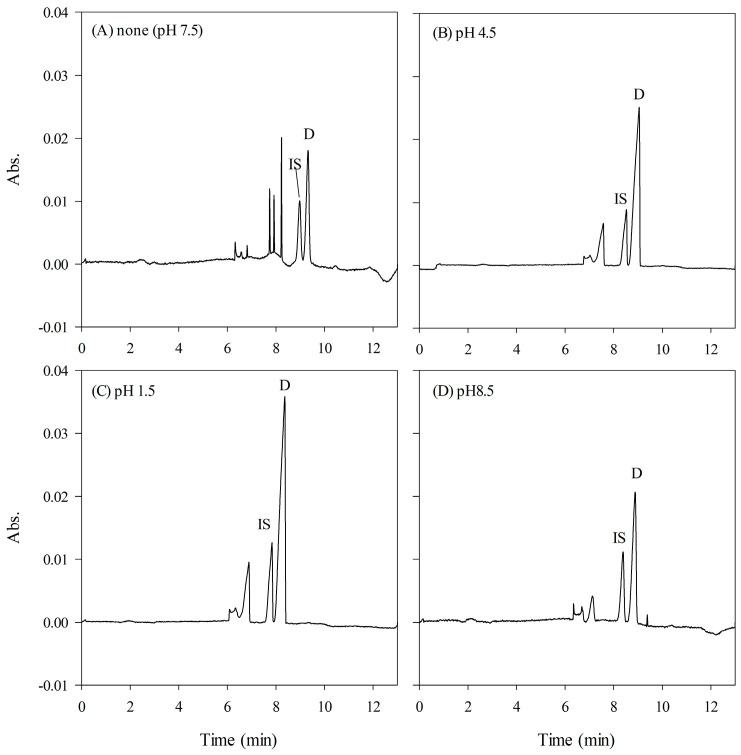
Effect of pH values of plasma on the SPE procedure. (**A**) pH 7.5, (**B**) pH 4.5, (**C**) pH 1.5, (**D**) pH 8.5. D: doripenem, 45 μg/mL; IS: internal standard, 10 μg/mL. CE conditions: HCB, 150 mM phosphate (pH 2.5) with 20% (*v/v*) of methanol; sweeping buffer, 50 mM phosphate (pH 2.5) with 100 mM SDS; sample injection, 5 psi, 80 s; separation voltage, −30 kV; detection wavelength, 300 nm.

Considering the hydrophilic nature of doripenem, traditional liquid–liquid extraction methods are deemed impractical for extracting these drugs from biological samples [34,35]. Despite the rigorous exploration of various solvent combinations, satisfactory extraction results remained elusive. Therefore, we opted to employ SPE, using Oasis^®^ HLB sorbent-based cartridges, as a preliminary sample preparation method. The extraction efficiency was found to be significantly influenced by the pH value of the plasma samples, owing to the zwitterionic nature of doripenem and meropenem, characterized by ionizable structures comprising a pyrrolidine ring and a carboxyl group. The corresponding pKa values for the carboxyl group and the N-atom on the pyrrolidine ring are provided in Table 1 [36]. Subsequently, a series of optimization experiments was conducted within the pH range of 1.5 to 8.5 to ascertain the optimal pH condition for SPE extraction. Gradually adjusting the sample pH to 1.5 revealed an augmentation in the recovery rate as the sample underwent increased acidification, indicative of an intensified interaction between the cationic analytes and the sorbents. Consequently, a final pH value of 1.5 was determined to be the optimal condition for SPE extraction (Figure 1).

### 2.2. The Concept of FESS-Sweeping MEKC Model

**Figure 2:**
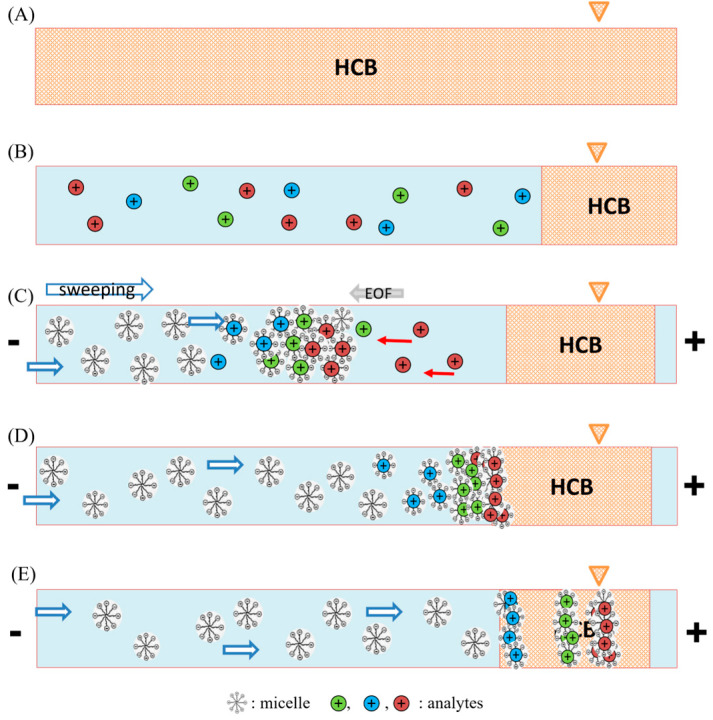
This schematic diagram by FESS-sweeping in MEKC. The detailed mechanism is discussed in the Results and Discussion section. (**A**) Fully flush the capillary with a high-conductivity buffer (HCB). (**B**) A long sample plug containing analytes prepared in a low-conductivity buffer is hydrodynamically injected. (**C**) The initial stage of stacking occurs under a negative voltage, where SDS micelles move toward the detector (blue-white arrow to the right) while sweeping and concentrating cationic analytes (reversely move to the cathodic inlet, red left arrow) in narrower bands, forming a sweeping boundary. (**D**) The second stacking stage involves additional analyte accumulation as the sweeping boundary approaches the HCB zone. (**E**) Separation performs in the MEKC mode.

The FESS-sweeping MEKC model was implemented within a low pH environment to efficiently suppress the counteractive electroosmotic flow (EOF) and enable the cationization of doripenem. Initially, the capillary was flushed with a high-conductivity buffer (HCB) (Figure 2A), followed by the hydrodynamic injection of a substantial sample plug (Figure 2B). The pretreated samples were reconstituted using a lower conductivity phosphate buffer. After that, both ends of the capillary were immersed in the sweeping buffer, and a negative voltage was applied. At this stage, anionic SDS micelles entered from the cathodic inlet of the capillary, effectively sweeping the reversed-moving cationic analytes toward the detector. This resulted in the formation of a sweeping boundary between the micelles and the analytes, indicating the initial phase of stacking, where the analytes were concentrated into narrower bands (Figure 2C). The second stage of stacking took place when the sweeping boundary approached the HCB zone. The existence of different conductivities between the sample solution and the HCB facilitated the additional accumulation of the analytes by reduced electric field strengths (Figure 2D). Ultimately, the separation process was carried out employing the MEKC mode, thereby enabling the efficient separation of the analytes (Figure 2E).

### 2.3. Optimization of FESS-Sweeping MEKC Model

Multiple parameters were examined to attain optimal sensitivity and separation efficiency, encompassing the pH value of the background solution, the organic modifier within HCB, the concentration of phosphate buffer, the injection duration of the sample plug, and the concentration of SDS. The separation voltage was established at a reverse polarity of 30 kV. 

#### 2.3.1. The pH Value of Background Solution

The modulation of pH in the buffer solution allows for control over the EOF and the dissociation behavior of the analytes. Under acidic conditions, the dissociation of silanol groups on the capillary wall can be suppressed, thereby further inhibiting the generation of EOF. Moreover, the analytes investigated in this study will also carry a positive charge. Different pH values (pH 2.0, pH 2.5, and pH 3.0) of phosphate buffer were studied. When a separation voltage of −30 kV was applied, the highest sensitivity and a stable current of 70 μA were achieved at pH 2.5. However, at a higher pH value of 3.0, the opposite EOF became stronger, leading to peak broadening. Conversely, at pH 2, currents of up to 120 μA and Joule heat generated an unstable baseline and poor reproducibility. Therefore, pH 2.5 was determined to be the optimal pH condition for the experiment.

#### 2.3.2. The Organic Modifier in HCB

**Figure 3:**
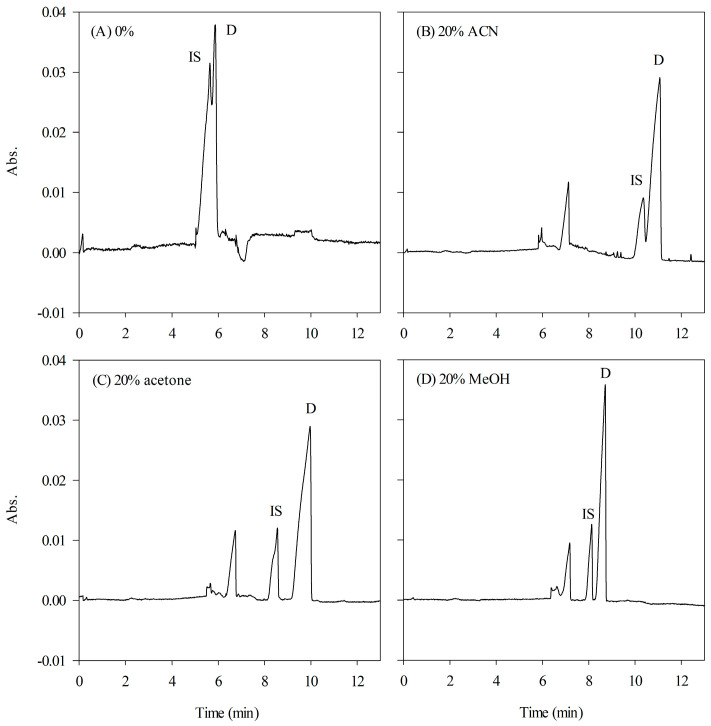
Effect of different organic solvents on HCB. (**A**) Absence of organic solvent, (**B**) ACN, (**C**) acetone, and (**D**) MeOH. The content of each organic solvent was 20%. See Figure 1 for other CE conditions.

**Figure 4:**
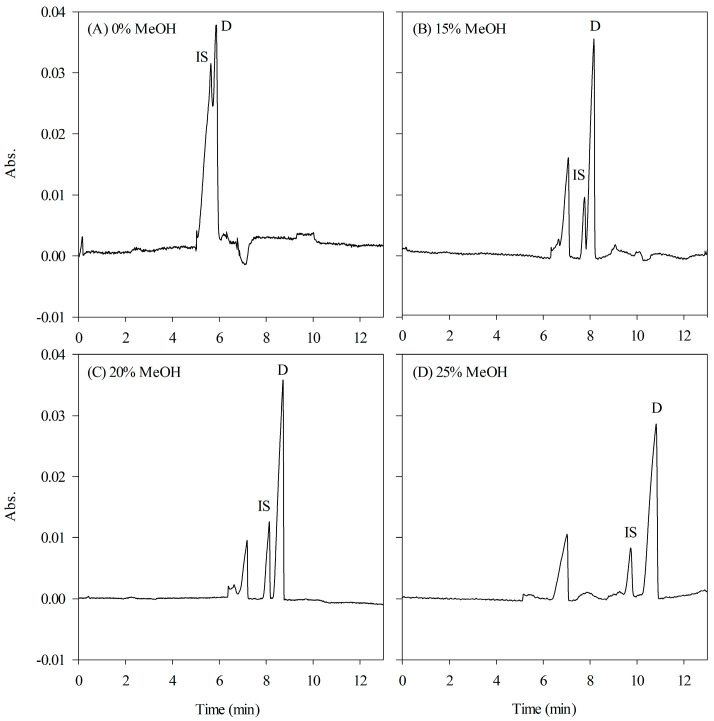
Effect of MeOH contents on HCB. (**A**) 0%, (**B**) 15%, (**C**) 20%, and (**D**) 25%. See Figure 1 for other CE conditions.

Adding organic solvents to the background buffer serves multiple purposes, including the adjustment of buffer polarity, which influences the partition coefficient of and selectivity of micelles, as well as the inhibition of EOF. Without an organic modifier, the analytes undergo efficient concentration during stacking but migrate together, resulting in a poor resolution. To improve the resolution, various organic solvents, such as methanol (MeOH), acetonitrile (ACN), and acetone, were added to the background buffer. As shown in Figure 3, incorporating 20% different organic modifiers into the HCB led to significant improvements in both resolution and peak heights. Among the tested organic solvents, MeOH exhibited superior performance of detection signals and peak symmetry. Figure 4 illustrates the impact of various concentrations of MeOH (ranging from 0 to 25%, *v/v*) on the HCB. Increasing the concentration of MeOH resulted in a progressive enhancement in the resolution; however, it also caused a delay in the migration time. Consequently, a concentration of 20% MeOH in the HCB was determined to be the optimal condition for further experimental investigations.

#### 2.3.3. The Phosphate Concentration in HCB and Sample Matrix

After sample pretreatment, we use phosphate buffer as the sample matrix for two primary reasons. Firstly, the analytes are introduced into the capillary along with the sample matrix through hydrodynamic injection. Therefore, electrolytes must be present in the sample matrix to avoid current leakage. Secondly, we aim to maintain consistency in the types of ions present within the separation system, ensuring the stability of the experiment. In addition, the nonuniform electric field strength between the HCB and the sample zone plays a vital role in the stacking effect of FESS. The analytes and micelles experience initial acceleration under the high electric field of the sample zone. In contrast, the HCB, with its high conductivity and low electric field, effectively reduces the electrophoretic velocities of both analytes and micelles. This variation in electrophoretic velocities between the zones enables the stacking of analytes into narrow bands, resulting in enhanced sensitivity.

**Figure 5:**
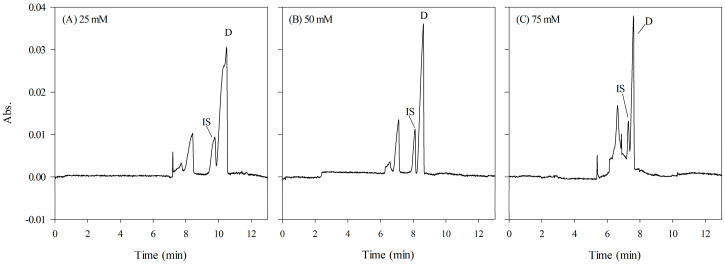
Effect of phosphate concentration in sample matrix. (**A**) 25 mM, (**B**) 50 mM, and (**C**) 75 mM. See Figure 1 for other CE conditions.

Different concentrations of phosphate were assessed in the HCB (100–200 mM, pH 2.5 with 20% MeOH, *v/v*) and the sample matrix (25–75 mM, pH 2.5) to determine the optimal stacking effect of FESS. Increasing the phosphate concentration in the HCB improved the resolution of analytes. However, at 200 mM phosphate, broadened signals and reduced sensitivity were observed due to the longitudinal diffusion of analytes and the generation of Joule heat. As for the phosphate concentration of the sample matrix, the conductivity difference between the HCB and the sample zone of 75 mM was insufficient. On the other hand, the overall electric field strength was much weaker at a diluted concentration of 25 mM. Decelerated electrophoretic velocities of analytes and inadequate interaction between analytes and micelles resulted in prolonged migration time and asymmetric shoulder peaks. Overall, the findings indicated that the phosphate concentration in the sample matrix had a greater influence on the FESS mode (Figure 5). Consequently, the optimal concentrations of phosphate buffer in the HCB and sample matrix were determined to be 150 mM and 50 mM, respectively.

#### 2.3.4. Time for Sample Injection and the Concentration of SDS

Although electrokinetic injection combined with sample stacking techniques offers higher achievable enrichment factors compared to hydrodynamic injection, it has the drawback of preferentially sampling higher mobility ions. This can amplify the negative impact of endogenous interferences in plasma, leading to issues like blocked capillaries and poor reproducibility, especially when the analytes have lower mobility than the sample matrix. In contrast, pressure injection provides an equal probability for all analytes. In this study, hydrodynamic injection was employed for sample injection. Injection times ranging from 70 to 90 s at 5 psi were investigated, resulting in sample lengths ranging from 70.39% to 90.5% of the effective length (50 cm). As expected, increasing the injection time resulted in enhanced sensitivity. However, longer injection times led to insufficient separation length in the capillary. Hence, the optimal injection time at 5 psi was determined to be 80 s. 

The sweeping technique involves the selective accumulation of analyte molecules via the pseudostationary phase (SDS micelles in this study) that infiltrates the sample zone during voltage application. The injected length of an analyte can be compressed by a factor of (1 + k), where k represents the retention factor of analytes. Higher retention factors can be achieved by increasing the concentration of SDS. Additionally, the differential partitioning of analytes between the aqueous phase and the micelle phase enables separation through the MEKC mode. This study investigated the effects of SDS concentrations ranging from 50 to 150 mM. While there was a slight reduction in the total analysis time at 150 mM SDS, higher concentrations resulted in poor resolution. Therefore, an optimal SDS concentration of 100 mM was determined.

### 2.4. Method Validation

The quantitative performance of the FESS-sweeping MEKC method was assessed by method validation based on the International Conference on Harmonization (ICH) guidelines [37], in terms of linearity, precision and accuracy, sensitivity, and selectivity. Meropenem served as the internal standard (IS) to ensure the method’s feasibility. The peak area ratio of doripenem was calculated by dividing the analyte’s peak area by that of the IS. The validation was conducted in plasma within concentrations ranging from 1 to 45 μg/mL.

#### 2.4.1. Linearity

Calibration curves were generated using five known concentrations of the analyte plotted on the X-axis against the peak area ratios on the Y-axis. Over the tested concentration range of 1 to 45 μg/mL, excellent coefficients of determination (r^2^ ≥ 0.9995) from intra- (n = 3) and inter-day (n = 5) analyses demonstrate the high linearity of the calibration curves (Table 2). Moreover, these curves covered the required clinical range, including the minimum inhibitory concentration (MIC) values and therapeutic ranges for doripenem against susceptible bacterial infections, highlighting its value in clinical applications [5].

#### 2.4.2. Accuracy, Precision, and Recovery

High, medium, and low levels of three different analyte concentrations were validated by spiking doripenem to blank human plasma. At each tested concentration, precision was assessed by the relative standard deviation (RSD) and accuracy by the relative error (RE). For the intra- and inter-day precisions, the RSD values were lower than 1.35% and 5.86%, respectively. The RE values were below 3.33% for the intra-day analysis and below 4.63% for the inter-day analysis. The precision and accuracy of the method were evaluated and found to be good. 

The extraction recoveries of doripenem in plasma after the sample pretreatment procedure were also examined at the concentrations of 5, 25, and 40 μg/mL. The extraction recoveries were between 71.35 and 72.99%.

#### 2.4.3. Selectivity and Specificity

Ten blank plasma samples from patients receiving doripenem treatment and healthy volunteers were collected for the selectivity test. Clear baselines were observed around the migration time of doripenem and the internal standard, indicating no endogenous interference. Moreover, common drugs such as antihypertensive drugs, antifungal agents, and penicillins were added to blank plasma for the specificity test. No signal interferences were observed under the proposed method. 

#### 2.4.4. Limit of Detection (LOD), Limit of Quantification (LOQ), and Sensitivity Enhancement

The limit of detection (LOD, S/N = 3) was estimated to be 0.4 μg/mL, and the limit of quantification (LOQ, S/N = 10) was 1 μg/mL, detected at 300 nm under optimized conditions. To obtain the enhancement factor of this method, we further compared MEKC (sampling at 0.5 psi, 5 s) with this FESS-sweeping MEKC method. After calculating the peak area of the analyte with FESS-sweeping divided into the peak area of the analyte with MEKC and multiplied by the dilution factor, a sensitivity enhancement of 488-fold was achieved. To the best of our knowledge, this CE method provides a lower LOD in plasma than the published HPLC-UV methods and also acts as an eco-friendly alternative analytical technique for the determination of doripenem in plasma [16].

### 2.5. Application

**Figure 6:**
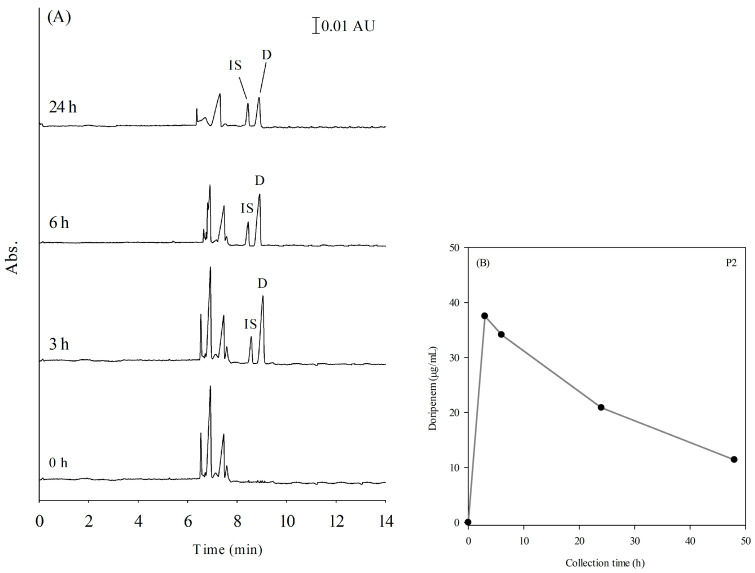
(**A**) Electropherograms of real plasma samples from patient 2 at different blood collection points. Other conditions are the same in Figure 1. (**B**) The trend of doripenem plasma concentrations at different collection points of P2.

We further applied the developed method to plasma samples collected from doripenem-treated patients before drug administration and during the dosing interval at five collection points. Since doripenem is an antibiotic of last resort for the treatment of multidrug-resistant bacterial infections, its plasma concentration is ideally maintained above the MIC during dosing intervals due to its time-dependent efficacy [10]. Apart from the clinical standard practice of intermittent intravenous (IV) administration, continuous IV infusions may be beneficial to maintain adequate drug concentrations and reduce fluctuations in plasma [11,38]. Patient 1 (P1) and patient 2 (P2) received doripenem 1.5 g per day by continuous IV infusions. Patient 3 (P3) and patient 4 (P4) were administrated doripenem by intermittent IV injection of 500 mg every 8 h and 250 mg every 12 h, respectively. Prior to CE analysis, the collected plasma samples were spiked with 15 uL of the internal standard (IS) and subjected to the pretreatment procedure described in Section 3.2. The electropherograms of a plasma sample from patient 2 (P2) are presented in Figure 6A. 

It is important to note that the presence of unknown peaks in the electropherogram can be attributed to the complexity of endogenous substances in patients’ plasma compared to that of healthy volunteers. The signal corresponding to doripenem was identified based on its migration time and confirmed using the standard addition method. A quantitative trend for patient 2 (P2) was obtained by plotting the doripenem plasma concentrations against the corresponding collection time points Figure 6B. Detailed information for patients P1 to P4 can be found in Table 3. The concentrations found in patients’ plasma were all within the linear range of this method. In addition, patients receiving continuous IV infusions of doripenem maintained plasma concentrations above MIC over 48 h with less fluctuation. In summary, the proposed FESS-sweeping method demonstrates good clinical applicability for analyzing plasma doripenem. 

## 3. Materials and Methods

### 3.1. Materials and Chemicals

Doripenem (D) and meropenem (used as internal standard, IS) were purchased from Sigma-Aldrich (St. Louis, MO, USA). Sodium dihydrogen phosphate monohydrate (NaH_2_PO_4_·H_2_O), sodium dodecyl sulfate (SDS), methanol (MeOH), sodium hydroxide (NaOH), acetonitrile (ACN), acetone, and hydrogen chloride (HCl) were all of analytical grade and purchased from Merck (Darmstadt, Germany). Milli-Q water from Millipore (Bedford, MA, USA) was used for preparing buffers and related aqueous solutions. Stock solutions were prepared in deionized water at 0.5 mg/mL for doripenem and 0.1 mg/mL for meropenem. These stock solutions were appropriately diluted with deionized water to obtain the desired analytical concentrations as working solutions. All solutions were stored in the refrigerator at 4 °C.

### 3.2. Pretreatment and Extraction of Plasma Samples

The plasma samples were collected from patients who had received doripenem, while blank plasma samples were obtained from healthy volunteers in our laboratory. This study was approved by the Institutional Review Board (CMUH105-REC1-083) at China Medical University. All blood samples were collected in plastic blood collection tubes containing heparin and centrifuged at 1500 rpm for 10 min at 4 °C. The resulting plasma, located in the upper layer, was then stored at −80 °C and thawed prior to analysis.

The blank samples (150 μL) were spiked with the doripenem stock solution to obtain the appropriate concentration for analysis, along with the IS working solution at a concentration of 10 μg/mL in plasma. Subsequently, the plasma samples were acidified to pH 1.5 using 1 M HCl and vortex mixed for 10 s. The SPE procedure was performed as follows: Firstly, a SPE column (Oasis^®^ HLB, Waters Corporation, Maple Street Milford, MA, USA) was prepared, which had been previously conditioned with 1 ml of methanol and subsequently equilibrated with 1 ml of water. Each acidified plasma sample was loaded onto the prepared SPE column. Once the sample was completely loaded onto the sorbent bed, the column was washed with 1.5 mL of water. The adsorbed solutes were then eluted with 1 mL of methanol and evaporated using a centrifugal vaporizer (EYELA CVE-200D, Tokyo, Japan) at 50 °C for 2 hours. The resulting residue was reconstituted with 40 μL of 50 mM phosphate buffer (pH 2.5) and filtered through a 0.45 μm PVDF filter (Millipore, Bedford, MA, USA) prior to CE analysis. 

### 3.3. CE System

All experiments were conducted using a Beckman P/ACE MDQ System equipped with a photodiode array detector and combined with the Beckman Coulter 32 Karat software (Fullerton, CA, USA) for instrument operation and data management. The UV absorbance wavelength was set at 300 nm. This CE method was performed using an uncoated fused-silica capillary (Polymicro Technologies, Phoenix, AZ, USA) with an inner diameter of 50 μm and an effective length of 50 cm (total length of 60 cm). Prior to analysis, new capillaries were pre-conditioned by flushing each solution for 10 min at 20 psi in the following sequence: MeOH, water, 1 M HCl solution, water, 1 M NaOH solution, and water. Between each run, the capillary was rinsed with MeOH for 5 min, 0.1 M NaOH for 5 min, water for 3 min, and running buffer for 7.5 min at 20 psi. 

### 3.4. FESS-Sweeping MEKC Model

The general electrophoresis procedures are described as follows. The capillary was thoroughly rinsed with the HCB, which consisted of 150 mM phosphate (NaH_2_PO_4_, pH 2.5) with 20% MeOH (*v/v*). After the HCB, a large volume sample with low conductivity was hydrodynamically introduced (5 psi, 80 s). The capillary inlet was then switched to the sweeping buffer, which contained 50 mM phosphate buffer (pH 2.5) with 100 mM SDS. Finally, the separation process began under an applied voltage of −30 kV.

## 4. Conclusions

We established a simple, sensitive, and robust FESS-sweeping MEKC method for the quantification of doripenem in human plasma. The method incorporated SPE pretreatment to ensure the effective extraction of doripenem while minimizing interference from the plasma matrix, resulting in a high recovery rate of 71.4%. Under optimized conditions, this method possessed excellent precision and accuracy in plasma. Moreover, the sensitivity of the developed method was significantly enhanced, which was increased by 488-fold compared with the conventional MEKC method. This method achieved an LOD of 0.4 μg/mL for doripenem and a short running time of less than 10 min. Plasma samples collected from patients receiving doripenem treatment had their doripenem concentrations successfully determined by the proposed method. Doripenem concentration at each collection point was within the range of the calibration curve, proving the applicability and reliability of this method. In conclusion, this FESS-sweeping MEKC method exhibits great potential and trustworthiness in clinical applications, which is an ideal alternative and environmentally friendly analytical tool for the determination of doripenem in plasma.

## Figures and Tables

**Table 1:** Structures and physicochemical properties of doripenem and meropenem.

(A) Doripenem		(B) Meropenem	
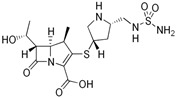	MW ^1^ = 438.52 pKa_1_ = 4.37; pKa_2_ = 9.39	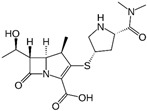	MW ^1^ = 383.46 pKa_1_ = 4.37; pKa_2_ = 8.34

^1^ MW, molecular weight (g/mol).

**Table 2:** Regression analysis, precision, and accuracy for determination of doripenem in intra-day (n = 3) and inter-day analysis (n = 5).

Doripenem	Linearity (1–45 μg/mL)		Precision and Accuracy		
	Regression Equation	r^2^	Spiked Conc. (μg/mL)	RSD (%)	RE (%)
Intra-day (n = 3)	y = (0.106 ± 0.0059) x + (0.0337 ± 0.0259)	0.9996	5.00 25.00 40.00	1.29 1.20 1.35	−3.01 3.33 2.05
Inter-day (n = 5)	y = (0.1087 ± 0.0037) x + (0.0394 ± 0.0394)	0.9995	5.00 25.00 40.00	5.86 1.17 1.96	−4.63 2.36 2.01

**Table 3:** Quantification of doripenem concentration in patients’ plasma.

Patient	Daily Dose	Dosage Interval	Collection Time Point (h)					Concentration Ranges (Premedication/Post Administration, μg/mL)
**Continuous administration**								
P1	1.5 g	1.5 g per day	0	4.5	6	24	48	0/15.06–39.00
P2	1.5 g	1.5 g per day	0	3	6	24	48	0/11.39–37.53
**Intermittent administration**								
P3	1.5 g	0.5 g per 8 h	0	1	2	6	8	0/9.37–44.97
P4	0.5 g	0.25 g per 12 h	0	0.75	2	4	8	0/9.64–44.86

## Data Availability

Not applicable.
